# From genes to epigenes: DNA methylation in the pathogenesis of Moyamoya disease

**DOI:** 10.17179/excli2025-8206

**Published:** 2025-03-10

**Authors:** Md Sadique Hussain, Vikas Jakhmola, Gyas Khan, Gaurav Gupta

**Affiliations:** 1 Uttaranchal Institute of Pharmaceutical Sciences, Uttaranchal University, Dehradun,Uttarakhand 248007, India; 2 Department of Pharmacology and Toxicology, College of Pharmacy, Jazan University, Jazan, Saudi Arabia; 3 Centre for Research Impact & Outcome-Chitkara College of Pharmacy, Chitkara University, Rajpura, Punjab 140401, India; 4 Centre of Medical and Bio-allied Health Sciences Research, Ajman University, Ajman, UAE

## ⁯⁯

Epigenetics examines genetic architecture and molecular modifications in chromatin that do not include variations in the arrangement of DNA bases. These modifications include mechanisms such as DNA methylation (DNAm), histone modification, and non-coding RNA regulation (Mattei et al., 2022[3]). DNAm involves the addition of a methyl group to the 5′-cytosine in CpG dinucleotides, affecting gene expression in response to external influences. Altered DNAm is a recognized molecular feature of several vascular disorders, exhibiting patterns that suggest common pathogenic mechanisms (Desiderio et al., 2024[1], Wang et al., 2024[6]). 

Recent research underscores the critical significance of epigenetic modifications, particularly DNAm, in the pathogenesis of Moyamoya disease (MMD). Sung et al. conducted a thorough investigation of gene activity and DNAm patterns in endothelial colony-forming cells (ECFCs) derived from three individuals with MMD as well as two healthy volunteers. Their investigation identified five candidate genes with elevated expression linked to hypomethylated promoter CpG sites (Sung et al., 2018[4]). Among them, Sortilin 1 (SORT1) emerged as a key gene to investigate due to its role in inhibiting endothelial vessel development and angiogenesis. The irregularities of SORT1 have been shown to influence multiple angiogenesis-related factors, suggesting a multifaceted role in vascular remodeling, a fundamental aspect of MMD.

He et al. expanded on these findings by using Illumina's 850K methylation panel to do a comprehensive whole-genome methylation analysis in ten individuals with ischemic MMD and 10 normal volunteers (He et al., 2022[2]). Their investigation identified significant hypermethylation, particularly in intergenic regions, which often serve as regulatory hubs for gene transcription. Among the 759 differentially expressed genes, SOX6 and RBM33 exhibited decreased expression, suggesting a possible link with vascular obstruction due to impaired endothelial integrity. Conversely, genes such as KCNMA1 and GALNT2 demonstrated increased activity, indicating their potential involvement in vascular restoration. Functional experiments confirmed these results, highlighting the complex interplay between hypoxia-induced vascular obstruction and compensatory angiogenesis.

To further advance the research, Tokairin et al. performed a similar methylation sequencing analysis using the Illumina 850K chip on two distinct female cohorts-one non-Asian (13 MMD individuals and 7 healthy controls) and the other Asian (14 MMD participants and 3 healthy controls) (Tokairin et al., 2024[5]). Their findings revealed a consistent decline in DNAm diversity across MMD participants from both ethnic groups. This observation was significant, as reduced methylation methylation variation correlated with impaired environmental adaptation and disrupted vascular homeostasis. Genes associated with critical physiological functions, including methylation, transcription regulation, restoration of DNA, cytoskeletal transformation, and natural killer cell signaling - played a substantial role in MMD. The cohort-based approach of the research emphasized that some epigenetic features could transcend racial disparities, potentially serving as universal markers of MMD. 

Furthermore, comparisons across these studies have begun to reveal converging pathways despite differences in methodology. The involvement of genes regulating angiogenesis, such as SORT1, KCNMA1, and GALNT2, suggests that aberrant DNAm affects not just the integrity of endothelial cells but also broader angiogenic pathways essential for MMD progression. Additionally, the identified intergenic hypermethylation may indicate a regulatory mechanism affecting enhancer regions, underscoring the importance of non-coding genomic elements in the disease pathology. 

This letter discusses research that demonstrates the effectiveness of advanced epigenomic innovations, such as Illumina methylation arrays, in elucidating the molecular underpinnings of MMD. They underscore the need for comprehensive multi-omics approaches - incorporating DNAm, transcriptomics, and proteomics - to achieve a more thorough understanding of disease mechanisms. Expanding sample sizes and including diverse populations in future investigation will be crucial for validating these findings and exploring potential racial or cultural influences.

While studies on DNAm in MMD are still evolving, identifying critical processes and genes offers valuable insights into disease progression. The interaction between epigenetic abnormalities and vascular remodeling presents an opportunity to explore innovative treatment approaches targeting these biological pathways. 

## Declaration

### Author contributions

Md Sadique Hussain: Data curation, writing - original draft. Vikas Jakhmola: Investigation, writing - original draft. Gyas Khan: Conceptualization, formal analysis, methodology. Gaurav Gupta: Conceptualization, formal analysis, writing - review & editing. 

All authors have approved the final version of the manuscript.

### Conflict of interest

The authors have no conflicts of interest.

### Using artificial intelligence (AI)

During the preparation of this work, the author(s) used ChatGPT to correct the grammatical and typographical errors in the manuscript. All authors have approved the final version of the manuscript.
